# Transcription regulator TRIP-Br2 mediates ER stress-induced brown adipocytes dysfunction

**DOI:** 10.1038/srep40215

**Published:** 2017-01-09

**Authors:** Guifen Qiang, Hyerim Whang Kong, Victoria Gil, Chong Wee Liew

**Affiliations:** 1 State Key Laboratory of Bioactive Substances and Functions of Natural Medicines, Institute of Materia Medica, Chinese Academy of Medical Sciences and Peking Union Medical College and Beijing Key Laboratory of Drug Target and Screening Research, Beijing, 100050, China; 2 Department of Physiology & Biophysics, College of Medicine, University of Illinois at Chicago, 835 S Wolcott Ave, M/C901, Chicago, IL, 60612, United States

## Abstract

In contrast to white adipose tissue, brown adipose tissue (BAT) is known to play critical roles for both basal and inducible energy expenditure. Obesity is associated with reduction of BAT function; however, it is not well understood how obesity promotes BAT dysfunction, especially at the molecular level. Here we show that the transcription regulator TRIP-Br2 mediates ER stress-induced inhibition of lipolysis and thermogenesis in BAT. Using *in vitro, ex vivo,* and *in vivo* approaches, we demonstrate that obesity-induced inflammation upregulates brown adipocytes TRIP-Br2 expression via the ER stress pathway and amelioration of ER stress in mice completely abolishes high fat diet-induced upregulation of TRIP-Br2 in BAT. We find that increased TRIP-Br2 significantly inhibits brown adipocytes thermogenesis. Finally, we show that ablation of TRIP-Br2 ameliorates ER stress-induced inhibition on lipolysis, fatty acid oxidation, oxidative metabolism, and thermogenesis in brown adipocytes. Taken together, our current study demonstrates a role for TRIP-Br2 in ER stress-induced BAT dysfunction, and inhibiting TRIP-Br2 could be a potential approach for counteracting obesity-induced BAT dysfunction.

Obesity, an epidemic affecting more than one third of the US population, is the result of an imbalance between energy intake and energy expenditure. In addition to its own specific morbidities, obesity is a major risk factor for metabolic conditions, such as insulin resistance, type 2 diabetes mellitus, cardiovascular diseases, and certain types of cancers^1^,^2^. Unlike white adipose tissue (WAT), which functions as the primary site for excess energy storage, brown adipose tissue (BAT) is known to play critical roles for both basal and inducible energy expenditure in the form of thermogenesis via the expression of the uncoupling protein 1 (UCP1)^3^. Increased BAT mass by transplantation has been shown to promote weight loss and improve systemic metabolism in models of both diet- and genetic-induced obesity^4^,^5^,^6^. On the other hand, obesity is associated with the reduction of BAT function^7^. However, it is not well understood how obesity promotes the dysfunction of BAT.

We^8^ and others^9^,^10^,^11^ have shown that adipocyte biology and function are tightly regulated by a complex network of transcription factors and coregulatory proteins. Previously, we showed that a transcriptional regulator, TRIP-Br2 (also known as SERTAD2), is specifically upregulated in both white and brown fat in obese mice and we further demonstrated that ablation of TRIP-Br2 protects mice from obesity and associated metabolic dysfunction^8^, supporting a role for TRIP-Br2 in adipocyte biology and energy metabolism. Further, our recent study observed that TRIP-Br2 plays a critical role in mediating endoplasmic reticulum (ER) stress-induced visceral fat inflammation^12^.

It is well established that obesity induces a state of chronic systemic low-grade inflammation originating from white adipose tissue^13^ and recent studies demonstrated that BAT from mice fed with high fat or cafeteria diet are also highly infiltrated with inflammatory macrophages^14^,^15^, suggesting a role for inflammation in obesity-induced BAT dysfunction. However, the molecular mechanism involved was not clearly defined. Here we show that obesity-induced inflammation upregulates TRIP-Br2 via the ER stress pathway in brown adipocytes and modulation of TRIP-Br2 expression significantly alters brown adipocyte lipolysis and thermogenesis. Our current study identifies TRIP-Br2 as a transcriptional mediator of obesity-induced BAT dysfunction.

## Results

### Brown adipocyte TRIP-Br2 expression is regulated by macrophage-secreted factors

**Figure 1:**
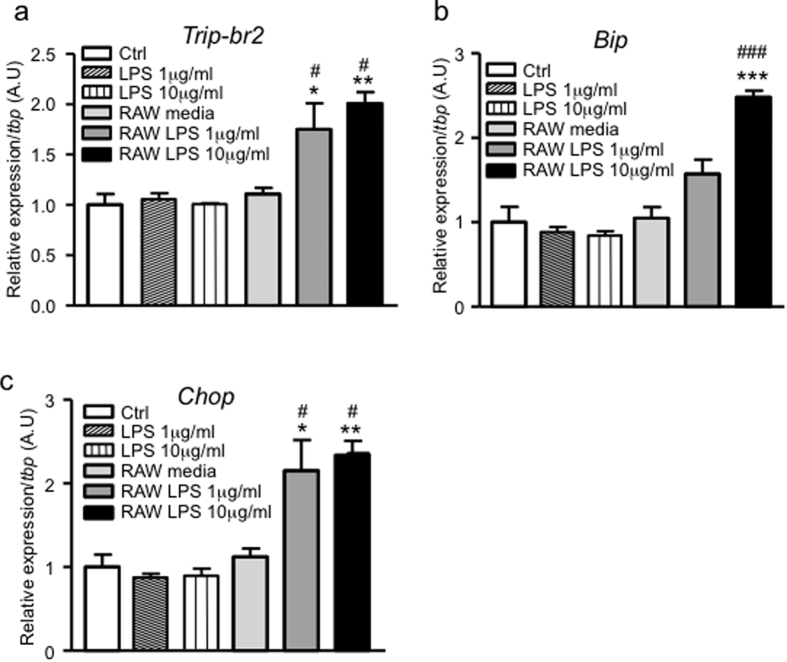
Brown adipocyte TRIP-Br2 expression is regulated by macrophage-secreted factors. qPCR analysis of TRIP-Br2 (**a**), BiP (**b**) or CHOP (**c**) gene expression in differentiated WT1 brown adipocytes treated with LPS alone (1 or 10 μg/ml) or RAW-conditioned media with or without LPS (1 or 10 μg/ml) stimulation for 24 h (n = 5). All qPCR data are normalized to TBP and presented as mean ± SEM. *p < 0.05; **p < 0.01; ***p < 0.001 (vs Ctrl); ^#^p < 0.05, ^###^p < 0.001 (vs RAW media).

Our previous study demonstrated that TRIP-Br2 is significantly upregulated in the brown fat of HFD-fed mice and ablation of TRIP-Br2 promotes energy expenditure and fatty acid oxidation in adipose tissues^8^. HFD induces accumulation and activation of immune cells, most prominently in the macrophages^15^,^16^,^17^ of both white and brown adipose tissues. To investigate whether macrophages play a role in the upregulation of TRIP-Br2 expression in brown fat, we treated differentiated brown adipocytes with lipopolysaccharides (LPS) or RAW (macrophage cell line) conditioned media with or without LPS stimulation. Interestingly, we observed that RAW-conditioned media, especially RAW cells stimulated with LPS, but not LPS alone, significantly upregulated *Trip-br2* expression in brown adipocytes (Fig. 1a).

We know that obesity induces systemic chronic low-grade inflammation and induction of ER stress by proinflammatory cytokines appears to be a contributing factor for the metabolic dysfunction in obese individuals^18^. To figure out whether macrophage-conditioned media-induced TRIP-Br2 expression is mediated by activation of the ER stress pathway, we examined the expression of ER stress markers. We observed that the upregulation of TRIP-Br2 expression could be due to induction of ER stress in differentiated brown adipocytes (Fig. 1b,c). Taken together, our data demonstrated that inflammation-induced ER stress modulates TRIP-Br2 expression in brown adipocytes.

### Pharmacologically-induced ER stress promotes TRIP-Br2 expression in brown adipocytes

**Figure 2:**
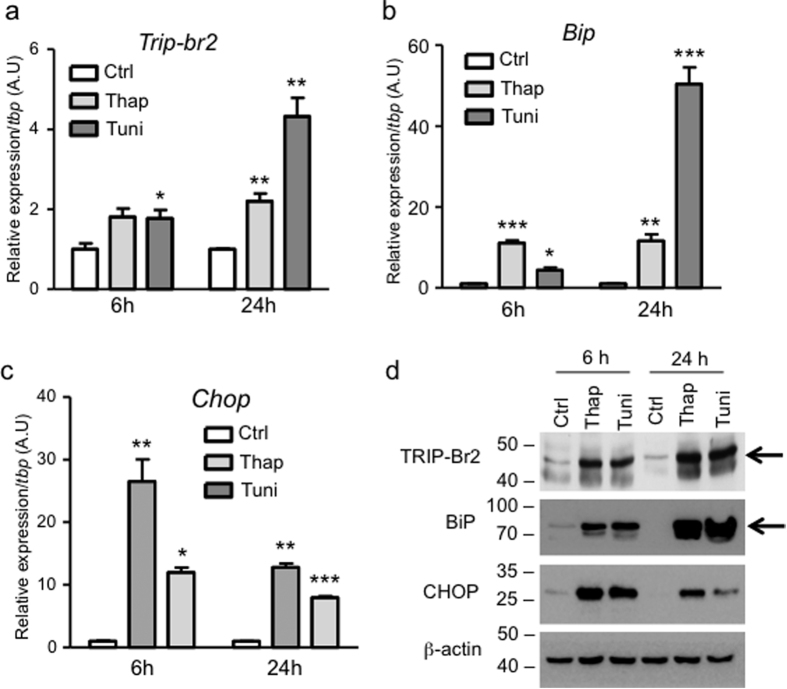
Chemically-induced ER stress upregulates TRIP-Br2 expression in brown adipocytes. qPCR analysis of TRIP-Br2 (**a**), ER stress markers BiP (**b**) or CHOP (**c**) gene expression in differentiated WT1 brown adipocytes after 6 or 24 h treatment with vehicle, thapsigargin (Thap, 0.1 μM) or tunicamycin (Tuni, 1 μg/ml) (n = 3); (**d**) Western blot for TRIP-Br2, BiP, CHOP or β-actin (loading control) protein in WT1 adipocytes treated with vehicle, thapsigargin (Thap, 0.1 μM) or tunicamycin (Tuni, 1 μg/ml) for 6 or 24 h. Uncut blots are included in the Supplementary information. All qPCR data are normalized to TBP and presented as mean ± SEM. *p < 0.05; **p < 0.01; ***p < 0.001.

In addition to ER stress induction, macrophage-secreted cytokines also activate cytokine signaling pathways leading to the regulation of other cellular process. To confirm that induction of TRIP-Br2 expression is indeed mediated by ER stress, we treated the differentiated brown adipocytes with two different pharmacological inducers of ER stress, thapsigargin and tunicamycin. We observed that both of the inducers significantly upregulated *Trip-br2* expression in a time-dependent manner, and tunicamycin appeared to be a stronger inducer than thapsigargin (Fig. 2a). The induction of ER stress was subsequently confirmed by ER stress markers expression (Fig. 2b,c). Using western blotting analysis, we confirmed that the transcriptional regulation of *Trip-br2* was accompanied by TRIP-Br2 protein upregulation (Fig. 2d).

**Figure 3:**
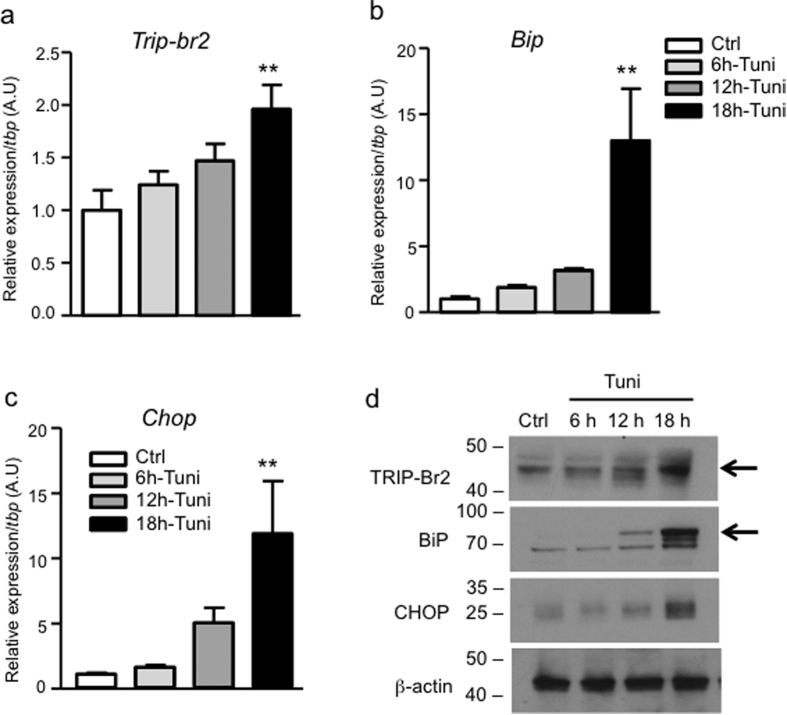
Tunicamycin-induced ER stress promotes TRIP-Br2 expression in brown adipose tissue. qPCR analysis of TRIP-Br2 (**a**), ER stress markers BiP (**b**), or CHOP (**c**) gene expression in brown adipose tissue harvested from mice 6, 12, or 18 h after intraperitoneal (IP) injection with vehicle or tunicamycin (Tuni, 2.5 mg/kg) (n = 5); (**d**) Western blot analysis of TRIP-Br2, BiP, CHOP, or β-actin (loading control) in brown adipose tissue from mice treated for 6, 12, or 18 h with vehicle or tunicamycin (Tuni, 2.5 mg/kg, i.p.). Uncut blots are included in the Supplementary information. All qPCR data are normalized to TBP and presented as mean ± SEM. *p < 0.05; **p < 0.01; ***p < 0.001.

To confirm the role of ER stress on TRIP-Br2 expression *in vivo*, we injected C57BL/6 (WT) mice with vehicle or tunicamycin. Brown adipose tissues were harvested for analysis after treatment for 6, 12, and 18 h. Similar to our *in vitro* observations, we found that tunicamycin significantly and time-dependently upregulated *Trip-br2* expression in brown fat (Fig. 3a). Induction of ER stress by tunicamycin *in vivo* in brown fat was confirmed by the increased expression of ER stress markers (Fig. 3b–d and Supplementary Fig. S1). Consistent with the gene expression data, we found upregulated protein expression of TRIP-Br2 and ER stress markers in brown fat after tunicamycin treatment (Fig. 3d).

### High-fat diet-induced ER stress induces TRIP-Br2 expression in brown fat

**Figure 4:**
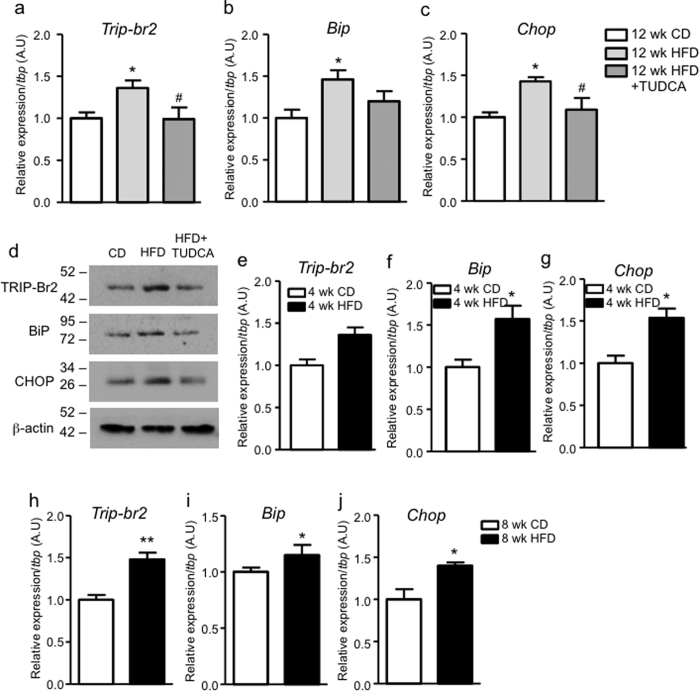
High-fat diet-induced obesity induces TRIP-Br2 expression in brown fat. qPCR analysis of TRIP-Br2 (**a**), ER stress markers BiP (**b**), or CHOP (**c**) gene expression in brown adipose tissue harvested from mice after 12 wk of CD, HFD, or HFD with TUDCA (i.p. 250 mg/kg at 8 am and 8 pm twice each day for 15 days) (n = 5); (**d**) Western blot analysis of TRIP-Br2, BiP, CHOP, or β-actin (loading control) in brown adipose tissue harvested from mice after 12 wk of CD, HFD, or HFD with TUDCA (i.p. 250 mg/kg at 8 am and 8 pm twice each day for 15 days). Uncut blots are included in the Supplementary information. qPCR analysis of TRIP-Br2 (**e**,**h**), ER stress markers BiP (**f**,**i**), or CHOP (**g**,**j**) gene expression in brown adipose tissue harvested from mice after 4 or 8 wk of CD or HFD (n = 5); All qPCR data are normalized to TBP and presented as mean ± SEM. *p < 0.05; **p < 0.01; ***p < 0.001 (vs Ctrl or CD); ^#^p < 0.01; ^##^p < 0.01 (vs HFD).

As tunicamycin is a chemical ER stress inducer, we next determined whether TRIP-Br2 is regulated by physiological ER stress, i.e. high-fat diet (HFD) feeding. Previously, we have demonstrated that after 12 wk of HFD, TRIP-Br2 expression is induced in brown adipose tissue^8^. To determine whether this is mediated by ER stress, we treated HFD-fed mice with the ER chaperone tauroursodeoxycholic acid (TUDCA)^19^. Our data showed that TUDCA treatment prevented the induction of *Trip-br2* expression, as well as ameliorated HFD-induced ER stress in brown adipose tissue (Fig. 4a–d and Supplementary Fig. S2). In addition, we also observed that *Trip-br2* expression was induced in brown fat even after 4 wk or 8 wk of HFD (Fig. 4e–g). Collectively, we showed that physiologically and pharmacologically-induced ER stress upregulates *Trip-br2* expression, confirming the link between ER stress and TRIP-Br2 regulation in brown fat.

### TRIP-Br2 mediates ER stress-induced inhibition of lipolysis in brown adipocytes

**Figure 5:**
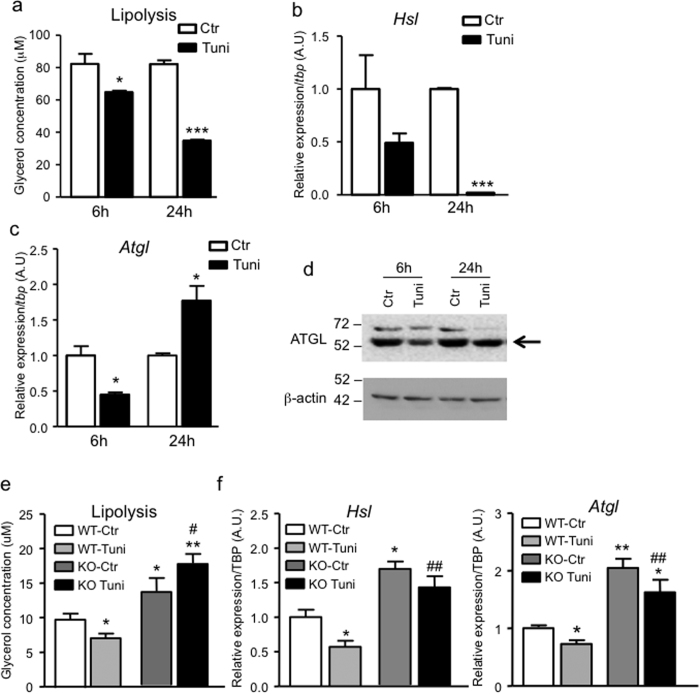
TRIP-Br2 mediates ER stress-induced downregulation of lipolysis in brown adipocytes. (**a**) Isoproterenol-stimulated lipolysis (1 μM for 2 h) in WT1 differentiated brown adipocytes treated with vehicle or tunicamycin (1 μg/ml) for 6 or 24 h (n = 3). qPCR analysis of (**b**) *Hsl* (**c**) *Atgl* gene expression in WT1 differentiated brown adipocytes treated with vehicle or tunicamycin (1 μg/ml) for 6 or 24 h (n = 3). *p < 0.05; ***p < 0.001 (vs Ctrl). (**d**) Protein expression of ATGL and β-actin (loading control) in WT1 differentiated adipocyte treated with vehicle or tunicamycin (1 μg/ml) for 6 or 24 h. Uncut blots are included in the Supplementary information. (**e**) Isoproterenol-stimulated lipolysis (1 μM for 2 h) in WT and TRIP-Br2 KO brown adipocyte treated with vehicle or tunicamycin (1 μg/ml) for 24 h (n = 6). (**f**) mRNA expression of *Hsl* and *Atgl* in WT and TRIP-Br2 KO brown adipocytes treated with vehicle or tunicamycin (1 μg/ml) for 24 h (n = 6). All qPCR data are normalized with TBP and presented as mean ± SEM. *p < 0.05; **p < 0.01; ***p < 0.001 (vs WT-Ctrl); ^#^p < 0.05; ^##^p < 0.01; ^###^p < 0.001 (vs WT-Tuni).

Since we have previously reported that ablation of TRIP-Br2 promotes visceral fat lipolysis^8^, to explore the effect of ER stress-induced TRIP-Br2 on the functional significance of brown adipocytes, in this study, we first examined the regulation of lipolysis in differentiated brown adipocytes after ER stress induction. Consistent with our previous study, we found that β-adrenergic agonist (isoproterenol)-stimulated lipolysis was significantly lower in tunicamycin-treated brown adipocytes in a time-dependent manner (Fig. 5a). To identify the potential mechanism, we first determined the gene expression of *Hsl* and *Atgl*, the key lipolytic enzymes. Indeed, we observed that the *Hsl* expression was downregulated after 6 h of treatment and almost undetectable after 24 h of treatment (Fig. 5b). Downregulation was also observed in *Atgl* expression after 6 h treatment, but upregulated after 24 h treatment (Fig. 5c). Upregulation of *Atgl* transcript could be a compensatory response to the downregulation of ATGL protein level in brown adipocytes treated with tunicamycin (Fig. 5d).

To investigate whether TRIP-Br2 plays a role in the ER stress-induced inhibition of lipolysis, we examined tunicamycin-treated WT and TRIP-Br2 KO differentiated primary brown adipocytes. We observed that ablation of TRIP-Br2 not only prevented ER stress-induced inhibition of lipolysis, but also enhanced isoproterenol-stimulated lipolysis, as previously observed in white adipocytes (Fig. 5e). This is likely due to upregulation of HSL and ATGL expression in the TRIP-Br2 KO brown adipocytes (Fig. 5f). Together, our results suggest that TRIP-Br2 mediates ER stress-induced inhibition of lipolysis.

### Ablation of TRIP-Br2 recovers the thermogenesis attenuated by ER stress in brown adipocytes

**Figure 6:**
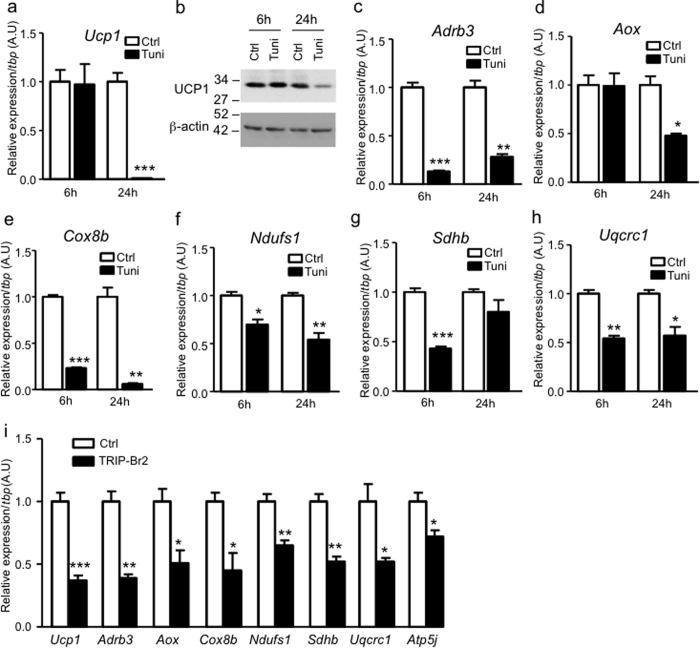
Ablation of TRIP-Br2 ameliorates the thermogenesis attenuated by ER stress in brown adipocytes. (**a**) mRNA expression (**b**) protein expression of UCP1 in WT1 differentiated adipocyte treated with vehicle or tunicamycin (1 μg/ml) for 6 or 24 h (n = 3). Uncut blots are included in the Supplementary information. mRNA expression of *Adrb3* (**c**), *Aox* (**d**), *Cox8b* (**e**), *Ndufs1* (**f**), *Sdhb* (**g**) and *Uqcrc1* (**h**) in differentiated WT1 adipocytes treated with vehicle or tunicamycin (1 μg/ml) for 6 or 24 h (n = 3). (**i**) mRNA expression of thermogenesis, fatty acid oxidation, and oxidative metabolism markers in differentiated brown adipocytes expressing Ctrl or TRIP-Br2 (n = 3). All qPCR data are normalized with TBP and presented as mean ± SEM. *p < 0.05; **p < 0.01; ***p < 0.001 (vs WT-Ctrl); ^#^p < 0.05; ^##^p < 0.01; ^###^p < 0.001 (vs WT-Tuni).

Since lipolysis, fatty acid oxidation, and thermogenesis are intimately linked^3^, we next examined whether inhibition of lipolysis would affect fatty acid oxidation and thermogenic processes in brown adipocytes. In line with the attenuated lipolysis, we observed that the thermogenesis was reduced, as shown by the thermogenic marker uncoupling protein 1(*Ucp1*). *Ucp1* expression was almost undetectable after 24 h tunicamycin treatment (Fig. 6a). This was later confirmed by measuring UCP1 protein expression via western blotting analysis (Fig. 6b). In addition to lipolysis, the downregulation of thermogenesis could be contributed by the downregulation of the beta-adrenergic receptor (*Adrb3*), fatty acid oxidation (*Aox*), and oxidative phosphorylation (*Cox8b, Ndufs1, Sdhb, Uqcrc1*), as shown by respective markers in the tunicamycin-treated brown adipocytes (Fig. 6c–h).

To determine whether upregulation of TRIP-Br2 in brown adipocytes during obesity would affect brown adipocyte functions, we inducibly overexpressed TRIP-Br2 in differentiated brown adipocytes. Our results showed that overexpression of TRIP-Br2 significantly suppresses the expression of markers of thermogenesis, fatty acid oxidation, and oxidative phosphorylation (Fig. 6i).

**Figure 7:**
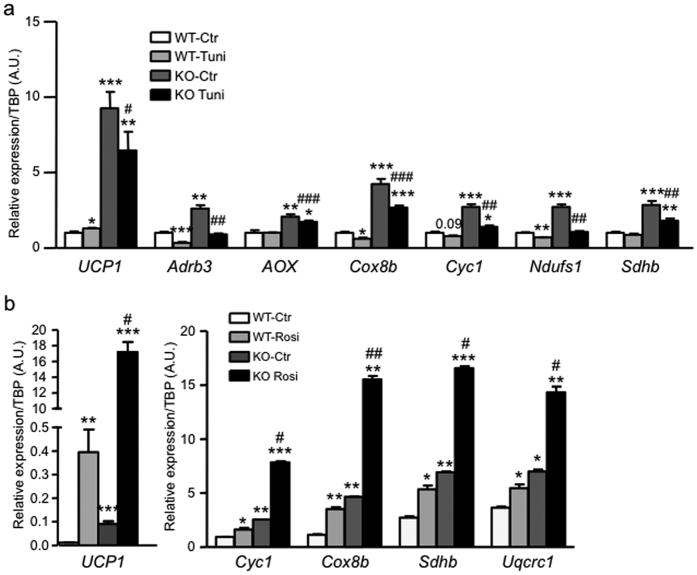
Ablation of TRIP-Br2 promotes brown adipocytes thermogenesis. (**a**) mRNA expression of thermogenesis, fatty acid oxidation, and oxidative metabolism markers in WT and TRIP-Br2 KO brown adipocytes treated with vehicle or tunicamycin (1 μg/ml) for 24 h (n = 4). (**b**) mRNA expression of thermogenesis, and oxidative metabolism markers in WT and TRIP-Br2 KO brown adipocytes treated with vehicle or Rosiglitazone (1 μg/ml) for 24 h (n = 3). All qPCR data are normalized with TBP and presented as mean ± SEM. *p < 0.05; **p < 0.01; ***p < 0.001 (vs WT-Ctrl); ^#^p < 0.05; ^##^p < 0.01; ^###^p < 0.001 (vs WT-Tuni or WT-Rosi).

When we examined differentiated primary brown adipocytes from WT and TRIP-Br2 KO, consistent with the metabolically protective phenotypes of the TRIP-Br2 KO mice^8^, we observed that under the basal state, ablation of TRIP-Br2 significantly upregulated the expression of markers of thermogenesis, fatty acid oxidation, and oxidative phosphorylation (Fig. 7a). In addition, we observed that despite inhibition by ER stress, the expression of markers of thermogenesis, fatty acid oxidation, and oxidative phosphorylation were still significantly higher in the TRIP-Br2 KO brown adipocytes than WT after tunicamycin treatment (Fig. 7a). To further examine the potential of TRIP-Br2 as a regulator of brown adipocyte functions, we treated differentiated primary brown adipocytes from WT and TRIP-Br2 KO mice with either vehicle or Rosiglitazone (1 μg/ml) for 24 h. Consistent with our findings so far, we observed that the absence of TRIP-Br2 in brown adipocytes significantly enhances Rosiglitazone-induced thermogenesis and its oxidative metabolism-promoting effects (Fig. 7b). Taken together, our findings suggest that TRIP-Br2 could potentially mediate downregulation of thermogenesis, fatty acid oxidation, and oxidative metabolism in brown adipocytes during obesity.

## Discussion

In this study, we observed that upregulation of TRIP-Br2 in brown adipose tissue during obesity is mediated by obesity-induced inflammatory cytokines via the ER stress pathway. Subsequently, we found that modulation of TRIP-Br2 expression significantly altered lipolysis, thermogenesis, fatty acid oxidation, and oxidative metabolism in brown adipocytes.

Obesity-induced inflammation and ER stress is known to negatively impact insulin sensitivity and adipocyte functions in WAT^20^; however, little is known about its effect on BAT. Until only recently, studies have shown that just like WAT, BAT is equally infiltrated with macrophages after diet-induced obesity (DIO)^14^. However, unlike WAT, the infiltrated macrophages were observed in the perivascular region in BAT, and not in crown-like-structures. Even though the exact role of perivascular macrophage accumulation in BAT is unclear, an increase in inflammatory cytokines in BAT after DIO has been clearly demonstrated^21^. In addition to the locally secreted inflammatory cytokines, as an extensively vascularized tissue, it is believed that BAT could also be affected by the elevated circulating inflammatory cytokines during obesity.

During obesity, in addition to the excess nutrient fluxes that activate the unfolded protein response, we and others have shown that proinflammatory cytokines also play determining and critical roles in the activation of ER stress in multiple metabolic organs, including white adipose tissues and liver^20^. This has not been investigated in BAT. In this study we showed that brown adipocytes are equally susceptible to ER stress induction upon cytokine stimulation. Our data clearly suggests that inflammation-induced ER stress potentially mediates BAT dysfunction during obesity.

Previously we have demonstrated that TRIP-Br2 is regulated by ER stress during obesity in visceral, but not subcutaneous, adipose tissue and upregulation of TRIP-Br2 is an important mediator for the ER stress-induced inflammation observed in visceral adipose tissue^12^. Unexpectedly, in the current study, we observed that obesity-induced ER stress also regulates TRIP-Br2 in brown adipose tissues. In addition, our preliminary studies also demonstrated that, inline with our previous observation in visceral fat, ER stress-induced TRIP-Br2 expression in brown adipocytes is also mediated by the transcription factor GATA3 (data not shown). These observations could suggest that subcutaneous fat might respond to HFD-induced dysfunction differently from visceral and brown fat.

Based on our previous study, we hypothesized that TRIP-Br2 could be a molecular mediator for BAT dysfunction during obesity. Indeed, we observed that ablation of TRIP-Br2 not only abolished ER stress-induced inhibition of brown adipocyte lipolysis, but also preserved brown adipocyte thermogenesis after induction of ER stress. Our data suggests that inhibition of TRIP-Br2 could potentially ameliorate obesity-induced brown fat dysfunction.

In addition to inflammation, obesity is also associated with morphological changes in BAT. Excessive fat deposition is often observed in obese mice leading to BAT whitening and dysfunction^22^; however, the mechanism contributing to the BAT whitening and fat accumulation has not been extensively investigated. Our previous study demonstrated a role for TRIP-Br2 in adipocyte lipolysis^8^. Dysregulated lipolysis could potentially lead to aberrant fat accumulation as demonstrated by the adipose tissue-specific ATGL KO mouse^22^. In contrast to visceral adipocytes^23^,^24^, we observed that activation of ER stress in brown adipocytes leads to downregulation of the key lipolytic enzymes, ATGL and HSL, as well as the beta-adrenergic receptor, causing an inhibition of lipolysis. Consistent with the role of TRIP-Br2 in visceral fat, we observed that ablation of TRIP-Br2 abolished the inhibitory effects of ER stress on lipolysis in brown adipocytes.

In contrast to WAT, BAT is important for both basal and inducible energy expenditure in the form of thermogenesis, mediated by the expression of the tissue-specific UCP1. It is believed that brown adipose tissue could directly affect whole-body metabolism, modify insulin sensitivity and modulate weight gain. As BAT mass decreases with obesity^25^, a decline in BAT function has been suggested to contribute to the impaired metabolism under this condition. While recent studies have shown that BAT transplantation improves metabolic parameters in obesity models^5^,^6^, most studies on BAT remain associative and the molecular mechanisms that contribute to obesity-linked BAT dysfunction are largely unknown. An activation of brown fat thermogenesis is directly modulated by a cascade of intimately coupled events including activation of β-adrenergic signaling, triglyceride lipolysis, fatty acid oxidation, and oxidative metabolism. Our study clearly demonstrated suppressive roles for TRIP-Br2 on thermogenesis and related metabolic processes. Further, ablation of TRIP-Br2 also preserved brown adipocyte function under ER stress induction to a certain extent.

Taken together, our study has identified TRIP-Br2 as a critical molecular regulator that mediates obesity-linked BAT dysfunction induced by ER stress. The role of TRIP-Br2 on BAT dysfunction in humans warrants future investigation.

## Methods

### Reagents

Thapsigargin, tunicamycin (A: 9.3%, B:24.33%, C:52.35%, D:12.57%), TUDCA, LPS, human insulin, isobutylmethylxanthine (IBMX), dexamethasone, T3, indomethacin, doxycycline, G418, Rosiglitazone and polybrene were from Sigma-Aldrich.

### Antibodies

Mouse polyclonal antibodies to TRIP-Br2 were raised against the recombinant protein of human TRIP-Br2 as previously described^8^. Anti-BiP (#3177), anti-CHOP (#2895), and anti-cleaved caspase 3 (#9661) antibodies were obtained from Cell Signaling. Anti-ATGL (55190-1-AP) and anti-β-actin (66009-I-Ig) antibodies were from Proteintech. Anti-UCP1 (ab10983) antibody was from Abcam.

### Animals

Mice were housed in environmentally controlled conditions with a 12-h light/dark cycle and had free access to standard rodent pellet food and water. The animal protocols were approved by the Institutional Animal Care and Use Committee (IACUC) of University of Illinois at Chicago. Animal care was given in accordance with institutional guidelines. All animal experiments were performed in accordance with relevant guidelines and regulations. Male C57BL/6J mice were obtained from the Jackson Laboratory (USA). 6 or 8-week-old C57BL/6J animals were used in our experiments. TRIP-Br2 KO mouse has been described previously^8^.

#### High fat diet treatment

6-wk-old male C57BL/6 mice were fed a high-fat diet (60% kcal from fat; Research Diet). After 10 wk of HFD, the HFD+TUDCA group received intraperitoneal injection of TUDCA twice a day (250 mg/kg at 8 am and 8 pm, total 500 mg/kg/day) for 15 days. Age-matched HFD group mice were administered with the same volume of vehicle. Age-matched chow diet control mice were also administered with vehicle.

Tunicamycin treatment: 10-wk-old male C57BL/6J mice were intraperitoneally injected with tunicamycin (2.5 mg/kg) dissolved in DMSO and diluted with PBS containing 100 mM glucose, or vehicle (PBS with DMSO and glucose).

### Plasmids

TRIP-Br2 was PCR-amplified and cloned into pENT-TRE plasmid and then into pSLIK-Neo lentivirus vector from Iain Fraser^26^ using the Gateway System (Invitrogen). All plasmids were sequence verified.

### Cell culture

Mouse brown preadipocyte cell lines (WT1) cells were grown in DMEM supplemented with 10%FBS, 100 units/ml penicillin and 100 ug/ml streptomycin and maintained at 37 °C with 5% CO_2_ atmosphere in a humidified incubator. WT1 cells were differentiated with differentiation cocktail (50 nM insulin, 100 nM T3, 0.125 mM Indomethacin, 0.5 mM IBMX and 5 μM dexamethasone in DMEM with 10% FBS) for 2 days before 4 days in medium supplemented with 50 nM insulin and 1 nM T_3_. ER stress was induced in cells by treatment with thapsigargin (0.1 μM) or tunicamycin (1 μg/ml) for the indicated time points.

### Conditioned medium

Supernatant from RAW cells treated with control or LPS (1 or 10 μg/ml) for 24 h was used for conditioned medium.

### Primary stromal-vascular fraction isolation and differentiation

BAT were dissected from 5-6-wk-old TRIP-Br2 WT and KO mice, minced, and digested with collagenase Type II (Worthington) (2 mg/ml in KRBA containing 125 mM NaCl, 4.74 mM KCl, 1 mM CaCl_2_, 1.2 mM KH_2_PO4, 1.2 mM MgSO4, 5 mM NaHCO_3_, 25 mM Hepes (pH 7.4), 3.5% BSA + 5.5 mM glucose) for 45 min with shaking at 37 °C^27^, then centrifuged at 1000 rpm for 5 min. The SVF pellet was washed twice with KRBA and once with DMEM complete medium. Then, the pellet was resuspended in 5 ml of DMEM complete medium and filtered over 70 μm filter adaptor. Filtered SVF was plated onto rat tail collagen-I (Invitrogen)-coated dish. For differentiation, confluent primary preadipocytes were differentiated with 50 nM insulin, 100 nM T_3_, 0.125 mM Indomethacin, 0.5 mM IBMX and 5 μM dexamethasone in DMEM/F12 media supplemented with 10% FBS for 2 days, followed by 4 days in medium supplemented with 50 nM insulin and 1 nM T_3_ with media change in between.

### Stable inducible cell line

Immortalized WT1 preadipocytes were infected with pSLIK-TRE-TAP-Neo or pSLIK-TRE-TAP-hTRIP-Br2-Neo. 24 h after infection, cells were selected with 1000 ng/ml of G418 for up to 2 wks. TAP or TAP-TRIP-Br2 expression was induced by addition of 1000 ng/ml of doxycycline for indicated time.

### Lipolysis assay

Differentiated WT1 adipocytes or primary mouse brown adipocytes were incubated with DMSO or tunicamycin (1 μg/ml) for 6 h or 24 h. Then, the adipocytes were starved for 1 h with Krebs-Ringer HEPES buffer (KRBH) (120 mM NaCl, 25 mM HEPES, 4.6 mM KCl, 1 mM MgSO_4_, 1.2 mM KH_2_PO_4_, and 1.9 mM CaCl_2_, pH 7.4) followed by stimulation with 1 μM isoproterenol in KRBH for 2 h. The supernatant was taken and extracellular glycerol was measured using AdipoLyze Lipolysis Detection Assay Kit (Lonza).

### RNA extraction and real time PCR

Total RNA was isolated from tissues and cells with the use of Trizol reagent (Invitrogen) and Direct-zol kit (Zymo Research). cDNA was prepared from 1 μg of total RNA using the High Capacity cDNA Reverse Transcription Kit (Invitrogen) with random hexamer primers, according to the manufacturer’s instructions. The resulting cDNA was diluted 10-fold, and a 1.5 μl aliquot was used in a 6 μl PCR reaction (SYBR Green, Bio-Rad) containing primers at a concentration of 300 nM each. PCR reactions were run in triplicate and quantitated using the Applied Biosystems ViiA^TM^7 Real-Time PCR system. Results were normalized to *TATA box binding protein (TBP*) expression and expressed as arbitrary units or fold change. Primer sequences listed in Supplementary Table 1.

### Western blotting

Total cell or tissue lysates (20–50 μg) were subjected to SDS−PAGE and blotting was performed as described^28^. Multiple exposures were used to ascertain signal linearity.

### Statistical analyses

All data are presented as the mean ± S.E.M. and were analyzed by unpaired two-tailed Student’s *t* test or analysis of variance, as appropriate. *P* < 0.05 was considered significant.

## Additional Information

**How to cite this article**: Qiang, G. *et al*. Transcription regulator TRIP-Br2 mediates ER stress-induced brown adipocytes dysfunction. *Sci. Rep.* **7**, 40215; doi: 10.1038/srep40215 (2017).

**Publisher's note:** Springer Nature remains neutral with regard to jurisdictional claims in published maps and institutional affiliations.

## Supplementary Material

Supplementary Information
