# Supplementary material for: Transcription regulator TRIP-Br2 mediates ER stress-induced brown adipocytes dysfunction
Source: Sci Rep. 2017 Jan 9;7:40215. doi: 10.1038/srep40215 (PMC5220316; doi:10.1038/srep40215)

Supplementary Information

Transcription regulator TRIP-Br2 mediates ER stress-induced brown adipocytes dysfunction

Guifen Qiang[1, 2], Hyerim Whang Kong[2], Victoria Gil[2], Chong Wee Liew[2]

[1]Current affiliation: State Key Laboratory of Bioactive Substances and Functions of Natural Medicines, Institute of Materia Medica, Chinese Academy of Medical Sciences and Peking Union Medical College, Beijing, 100050, China

[2]Department of Physiology & Biophysics, College of Medicine, University of Illinois at Chicago, 835 S Wolcott Ave, M/C901, Chicago, IL, 60612, United States

Supplementary Figure Legends

Supplementary Figure S1

qPCR analysis of ER stress markers (a) *Xbp1s* (b) *Creb3*, (c) *Atf4* and (d) *Atf6* gene expression in brown adipose tissues harvested from mice 6, 12 or 18 h after intraperitoneal (IP) injection with vehicle or tunicamycin (2.5 mg/kg)(n=5). All qPCR data are normalized with TBP and presented as mean ± SEM. *,p<0.05; **,p<0.01; ***,p<0.001 (vs Ctrl).

Supplementary Figure S2

qPCR analysis of ER stress markers (a) *Xbp1s* (b) *Creb3*, (c) *Atf4* and (d) *Atf6* gene expression in brown adipose tissues harvested from mice after 12 wk of CD, HFD, or HFD with TUDCA (250 mg/kg at 8am and 8pm, i.p., total 500 mg/kg for 15 days) (n=5). All qPCR data are normalized with TBP and presented as mean ± SEM. *,p<0.05; **,p<0.01; ***,p<0.001 (vs CD); $^{\#}$,p<0.05; $^{\#\#\#}$,p<0.001 (vs HFD).

Supplementary Table 1

Primer sequences used in this study		
Gene		***Primer sequence***
mTrip-br2	Forward: 5'-	ATA TAT GTT GGG TAA AGG AGG AA -3'
	Reverse: 5'-	TGG CGC TGT AAG GTG TAA GAC -3'
mBip	Forward: 5'-	TTC AGC CAA TTA TCA GCA AAC TCT -3'
	Reverse: 5'-	TTT TCT GAT GTA TCC TCT TCA CCA GT -3'
mChop	Forward: 5'-	CCA CCA CAC CTG AAA GCA GAA -3'
	Reverse: 5'-	AGG TGA AAG GCA GGG ACT CA -3'
mTbp	Forward: 5'-	ACC CTT CAC CAA TGA CTC CTA TG -3'
	Reverse: 5'-	ATG ATG ACT GCA GCA AAT CGC -3'
mAtgl	Forward: 5'-	TAGCTAACAGTTGGGCTTCAC
	Reverse: 5'-	CAGAGAGAACAGAGCAGCTTAC
mHsl	Forward: 5'-	ACGGATACCGTAGTTTGGTGC
	Reverse: 5'-	TCCAGAAGTGCACATCCAGGT
mUcp1	Forward: 5'-	CTGCCAGGACAGTACCCAAG
	Reverse: 5'-	TCAGCTGTTCAAAGCACACA
mAdrb3	Forward: 5'-	GCTCTGTGTCTCTGGTTAGTTT
	Reverse: 5'-	GTCCAAGATGGTGCTTAGAGAG
mAox	Forward: 5'-	TCCTTAAACACCCACCCACCAAGA
	Reverse: 5'-	AAGAAAGGGAAGGTCAGCCACCAT
mCytc	Forward: 5'-	GCTACCCATGGTCTCATCGT
	Reverse: 5'-	CATCATCATTAGGGCCATCC
mCox8b	Forward: 5’-	GAACCATGAAGCCAACGACT
	Reverse: 5’-	GCGAAGTTCACAGTGGTTCC
mNdufs1	Forward: 5'-	CTTCAGGGAGGCATTCATTCT
	Reverse: 5'-	ATTGCTGCTCCATCATCTCTC
mSdhb	Forward: 5'-	CTGCCACACCATCATGAACT
	Reverse: 5'-	CTTGTAGGTCGCCATCATCTTC
mUqcrc1	Forward: 5'-	CAGATCGAGAAGGAACGAGATG
	Reverse: 5'-	GTCGCATGCAGGTAATCAAAG
mAtp5j	Forward: 5'-	CTCTTCGTGGACAAGATAAGAGAG
	Reverse: 5'-	CTCTGTCCAGATCTTGCTGATAC

Supplementary Figure S1

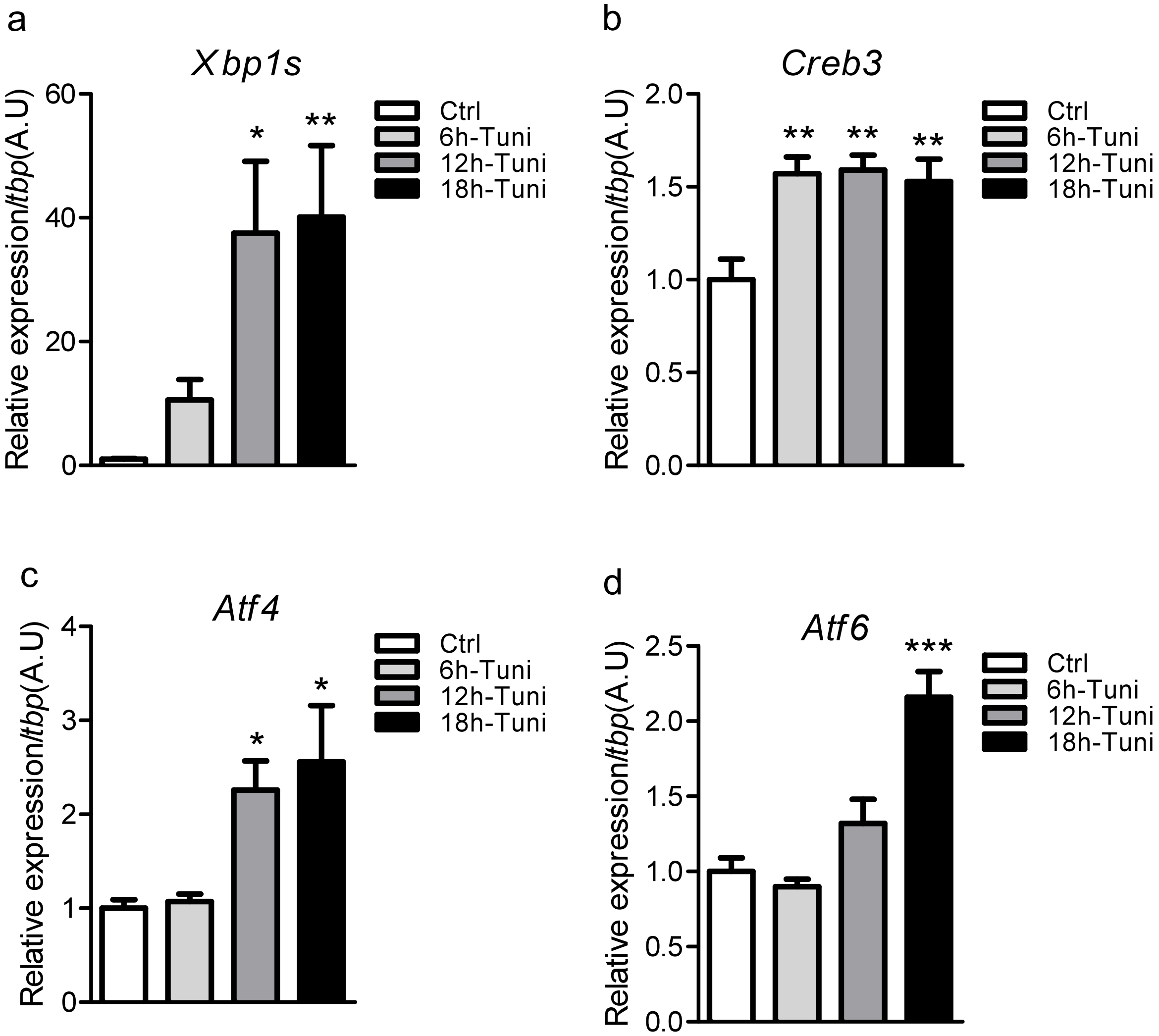

Suplementary Figure S2

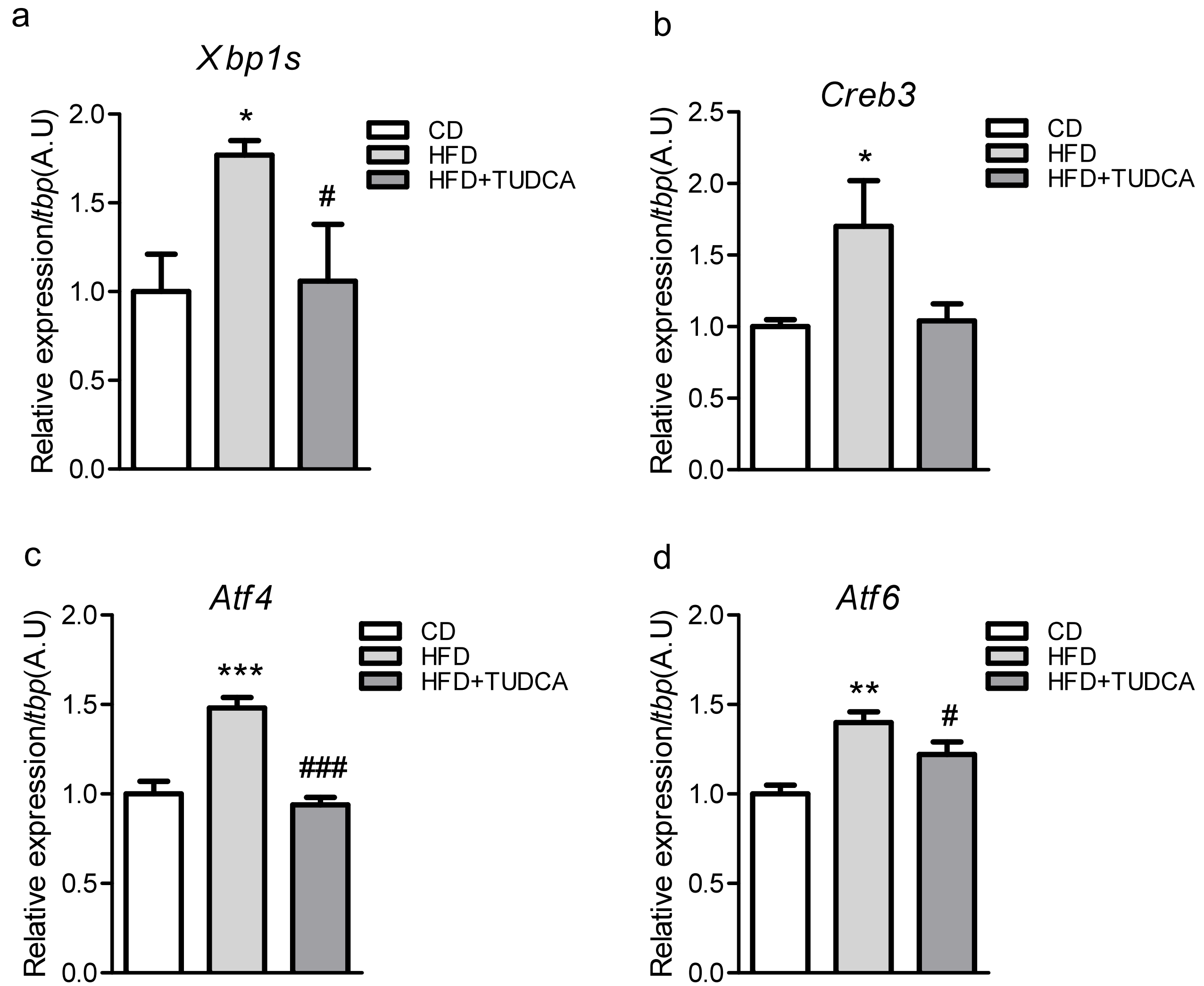

Uncut blot for Figure 2d

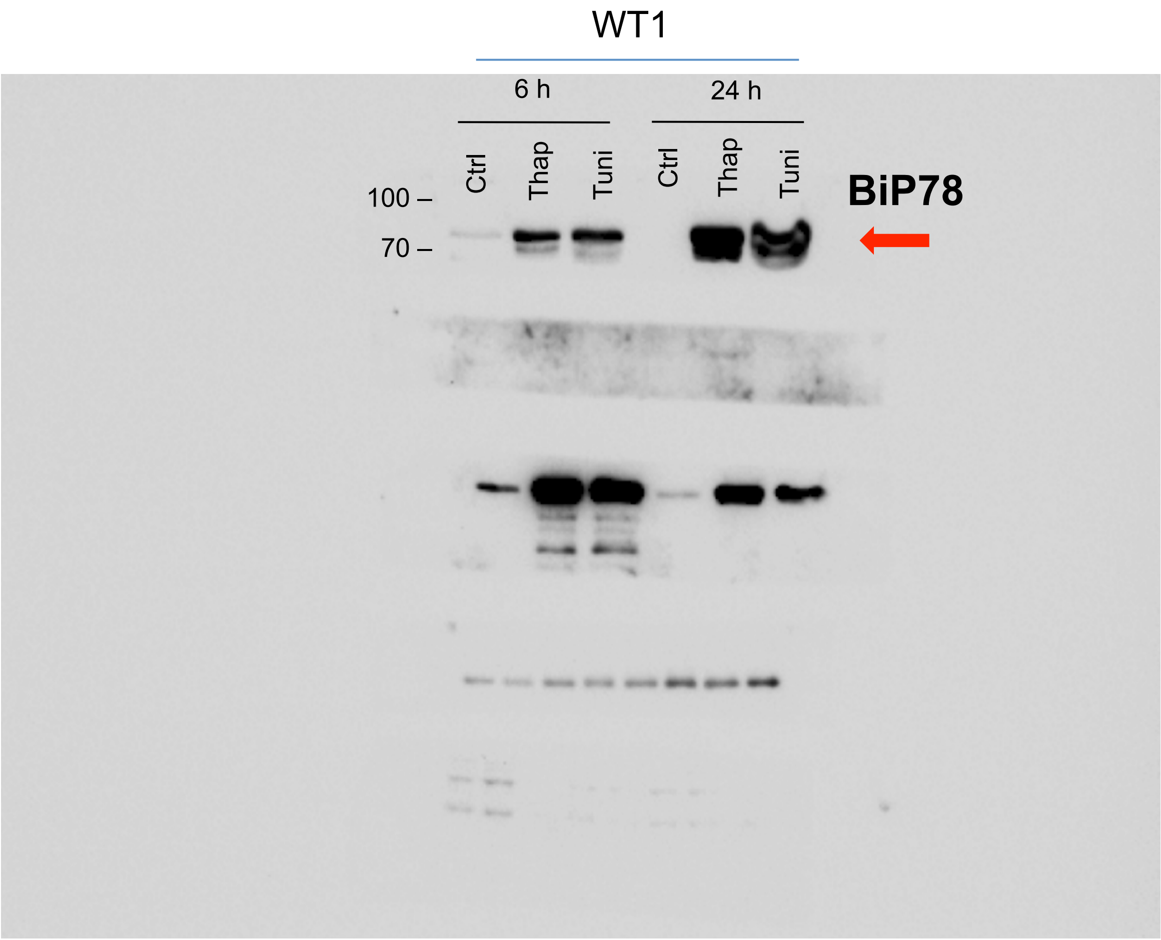

Uncut blot for Figure 2d

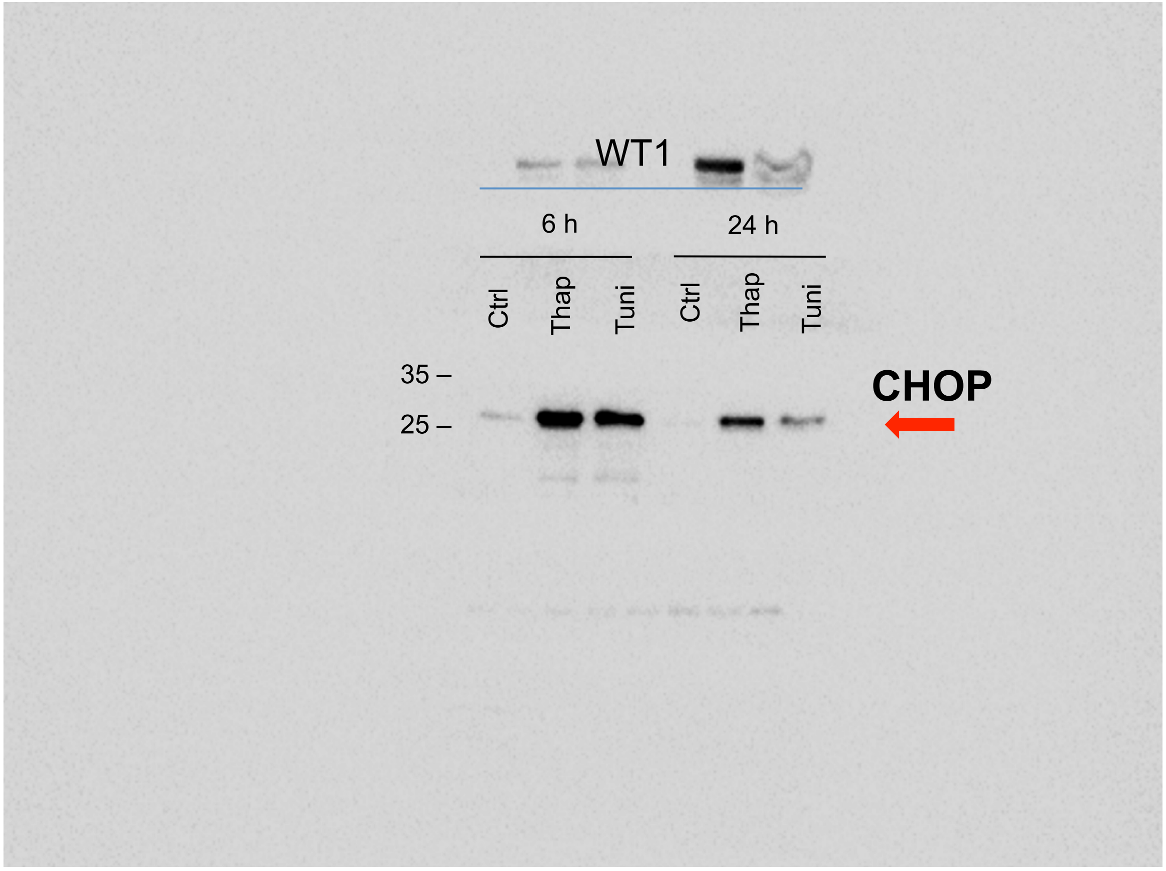

Uncut blot for Figure 2d

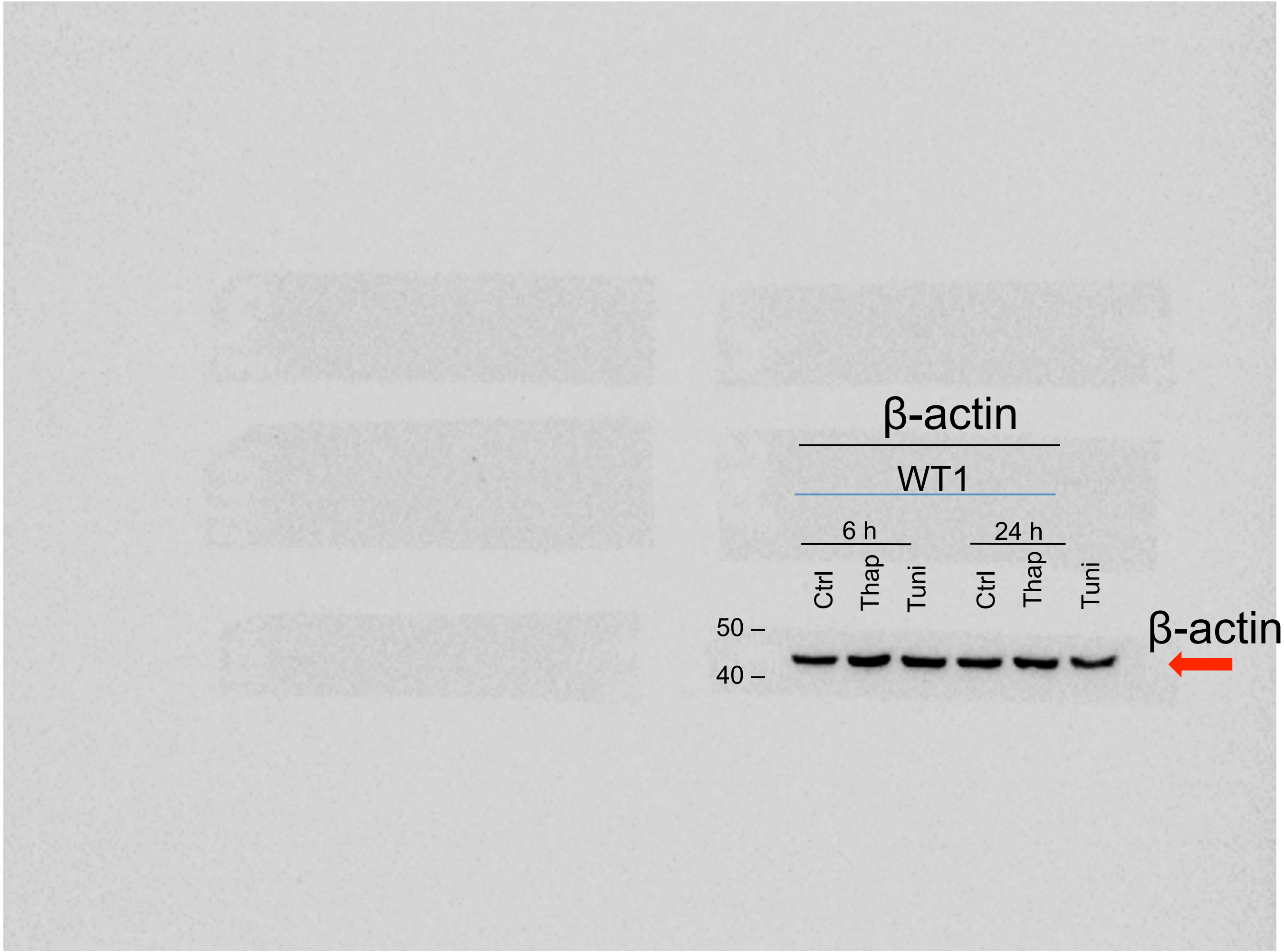

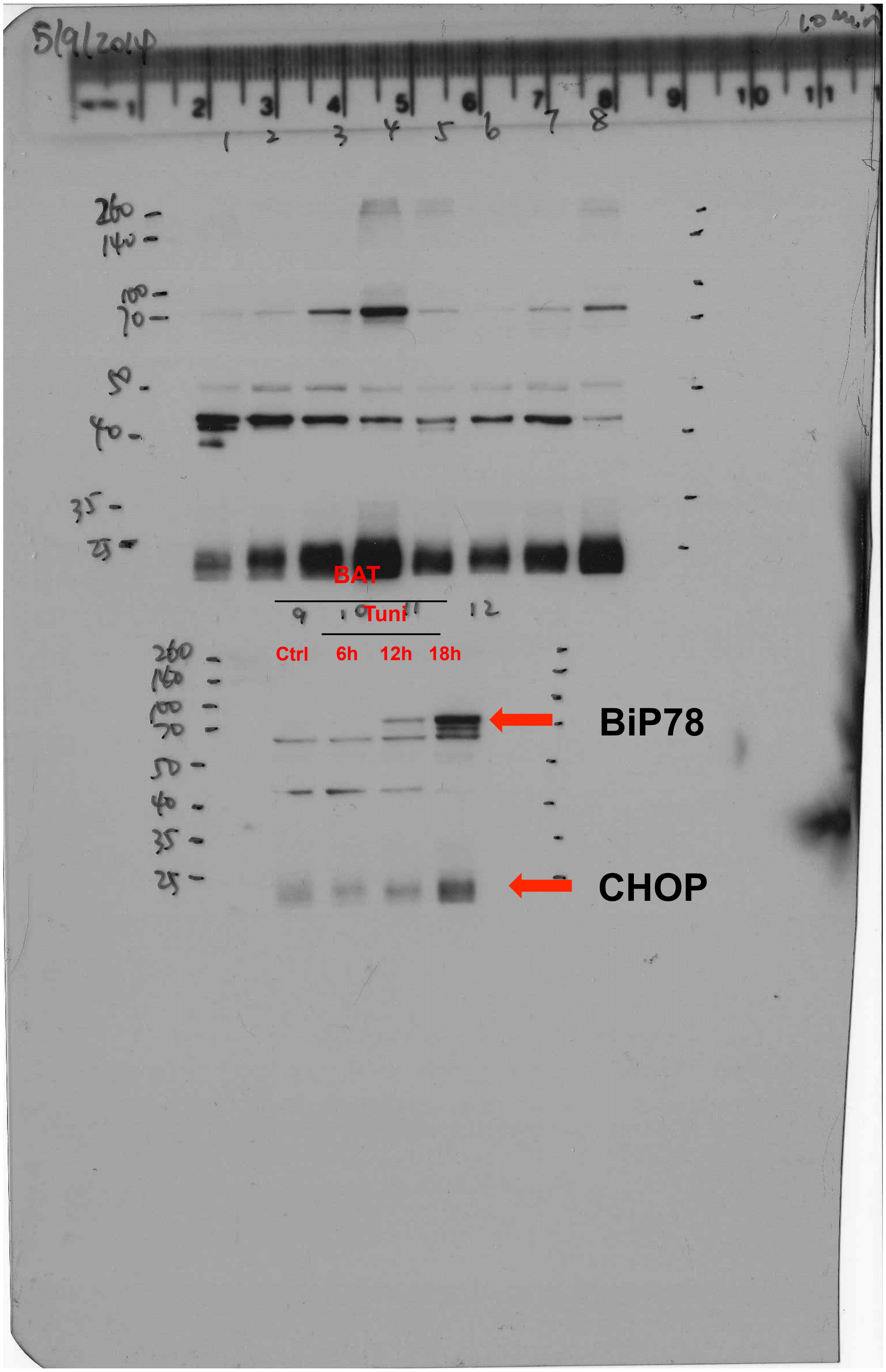
BAT
Tuni
Ctrl
6h
12h
18h
BiP78
CHOP

Uncut blot for Figure 3d

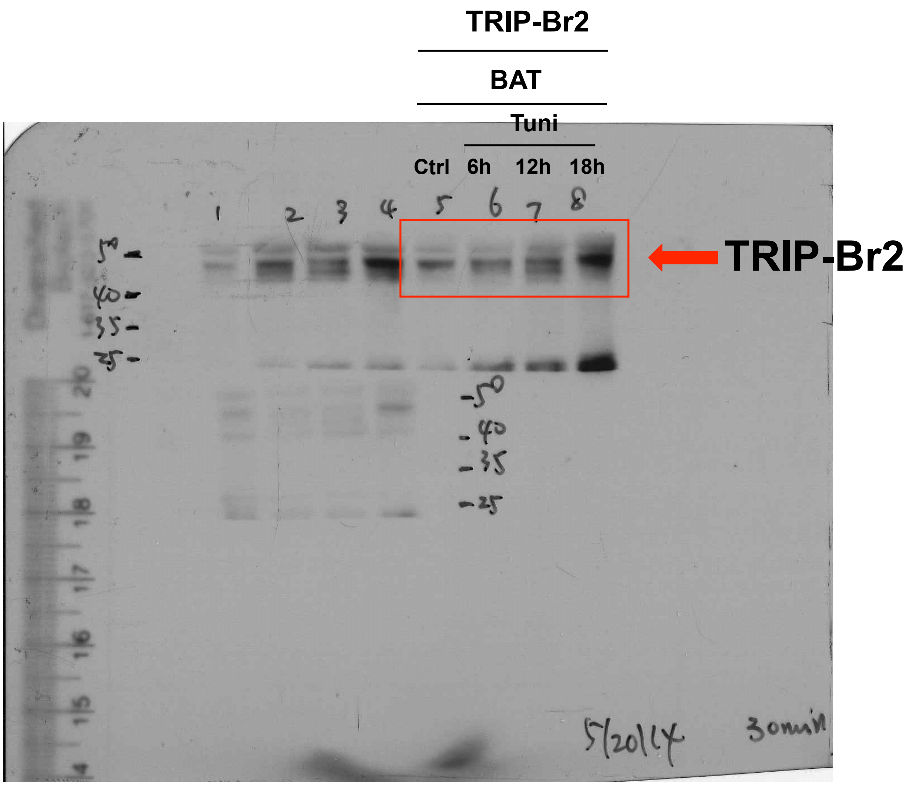

Uncut blot for Figure 3d

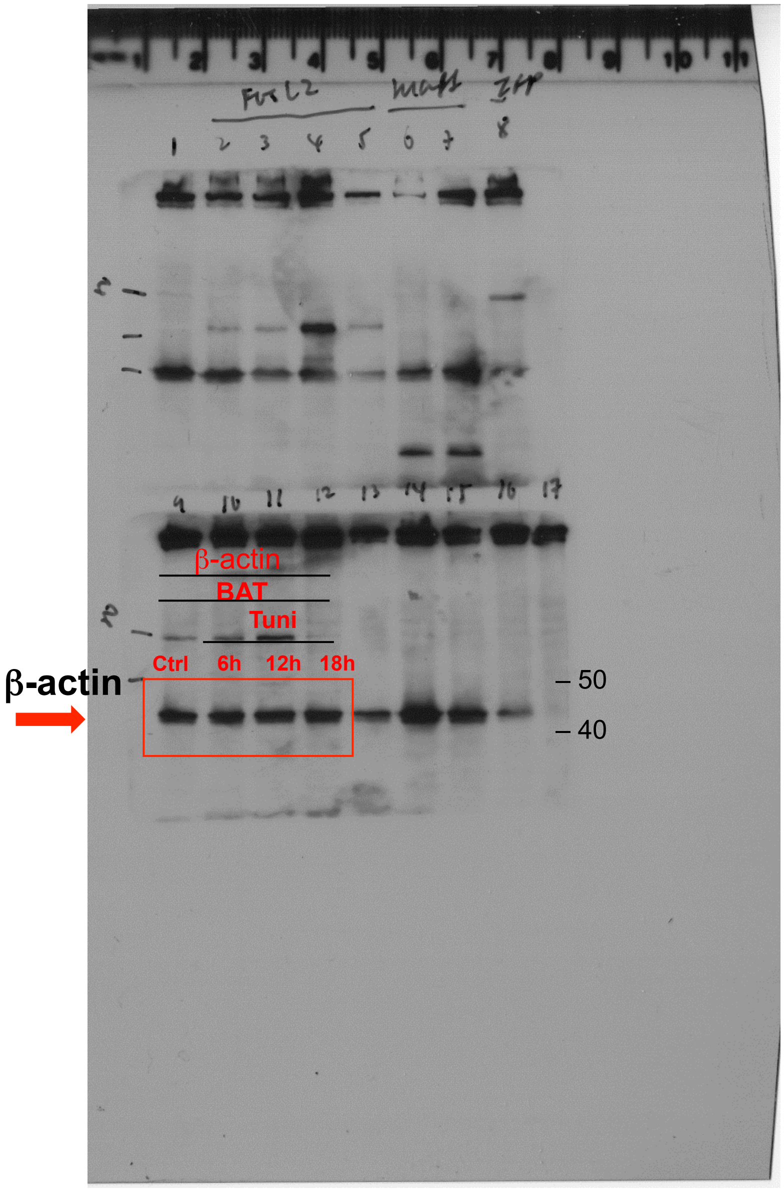

Uncut blot for Figure 4d

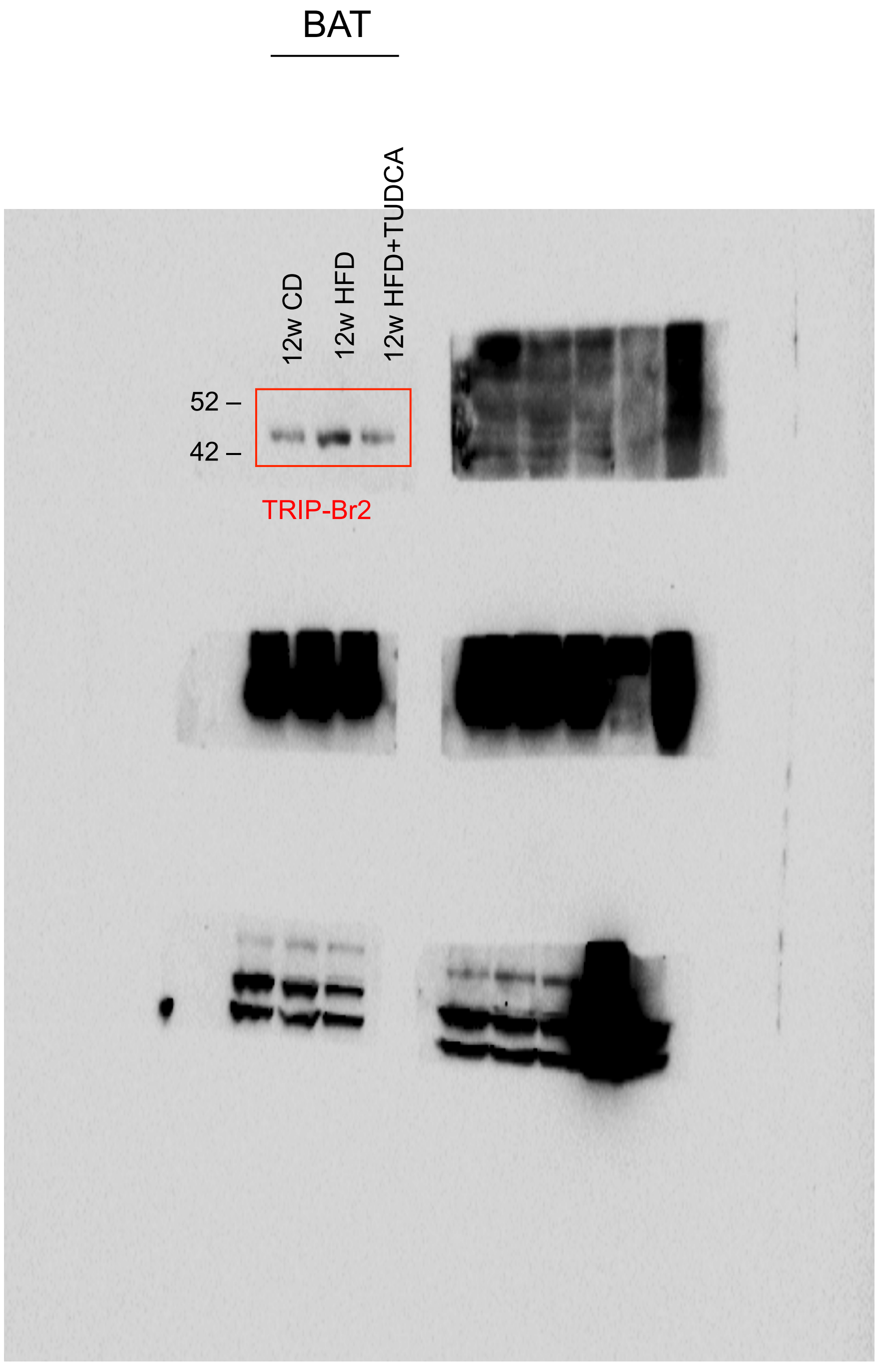

Uncut blot for Figure 4d

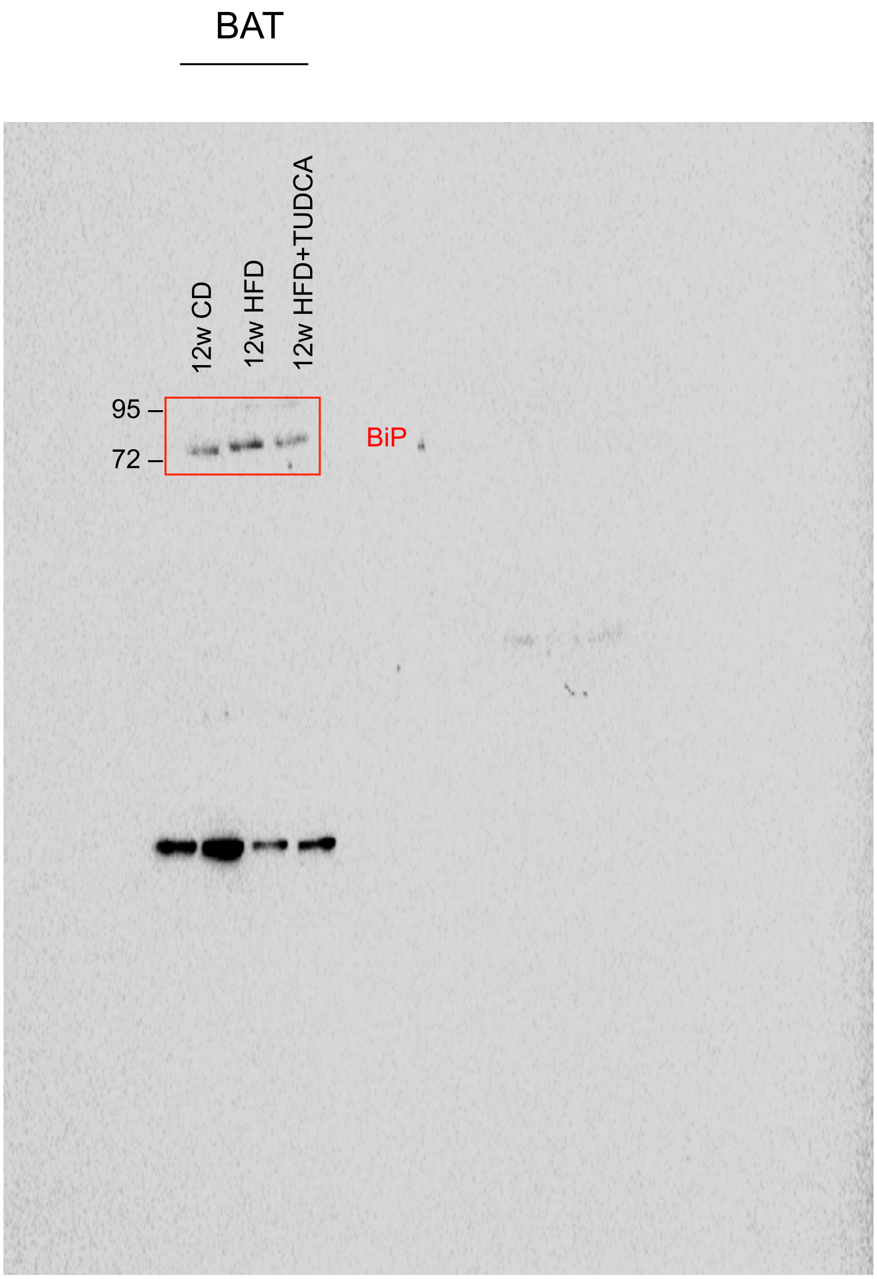

Uncut blot for Figure 4d

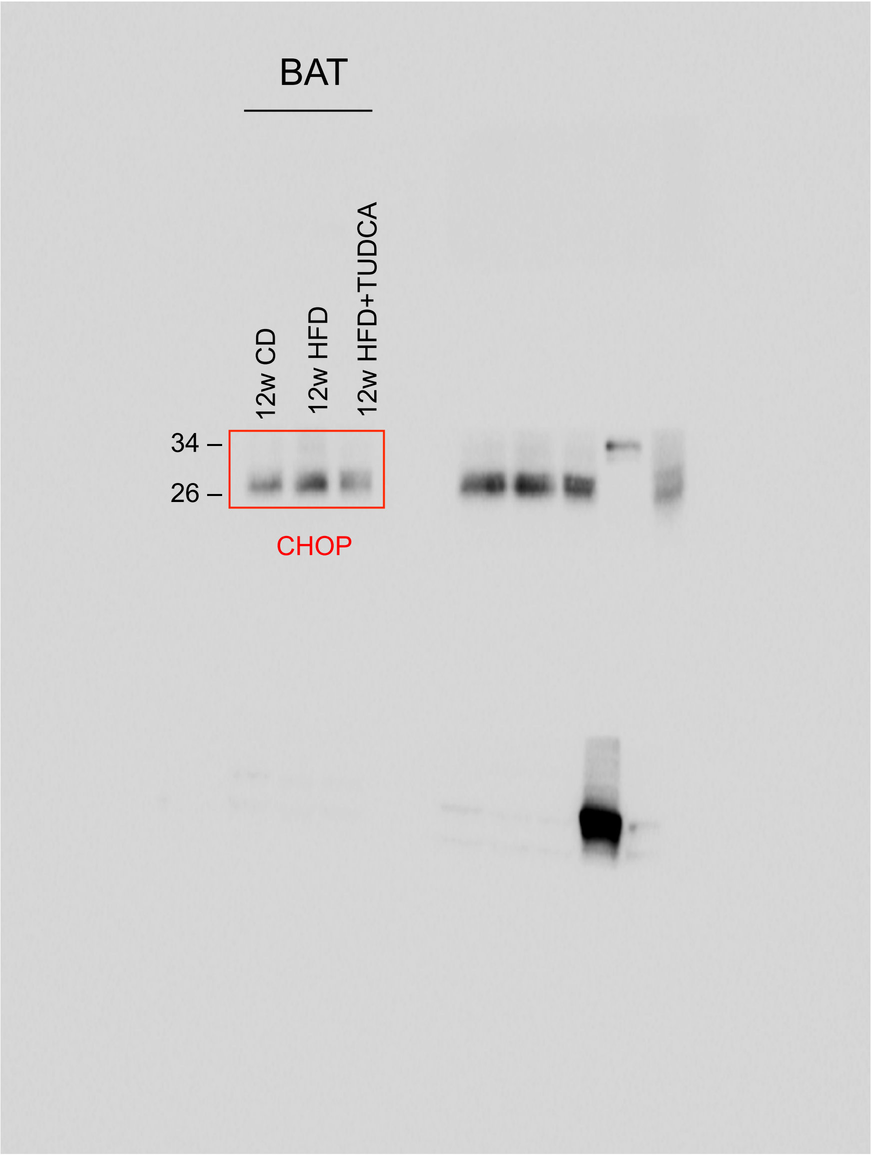

Uncut blot for Figure 4d

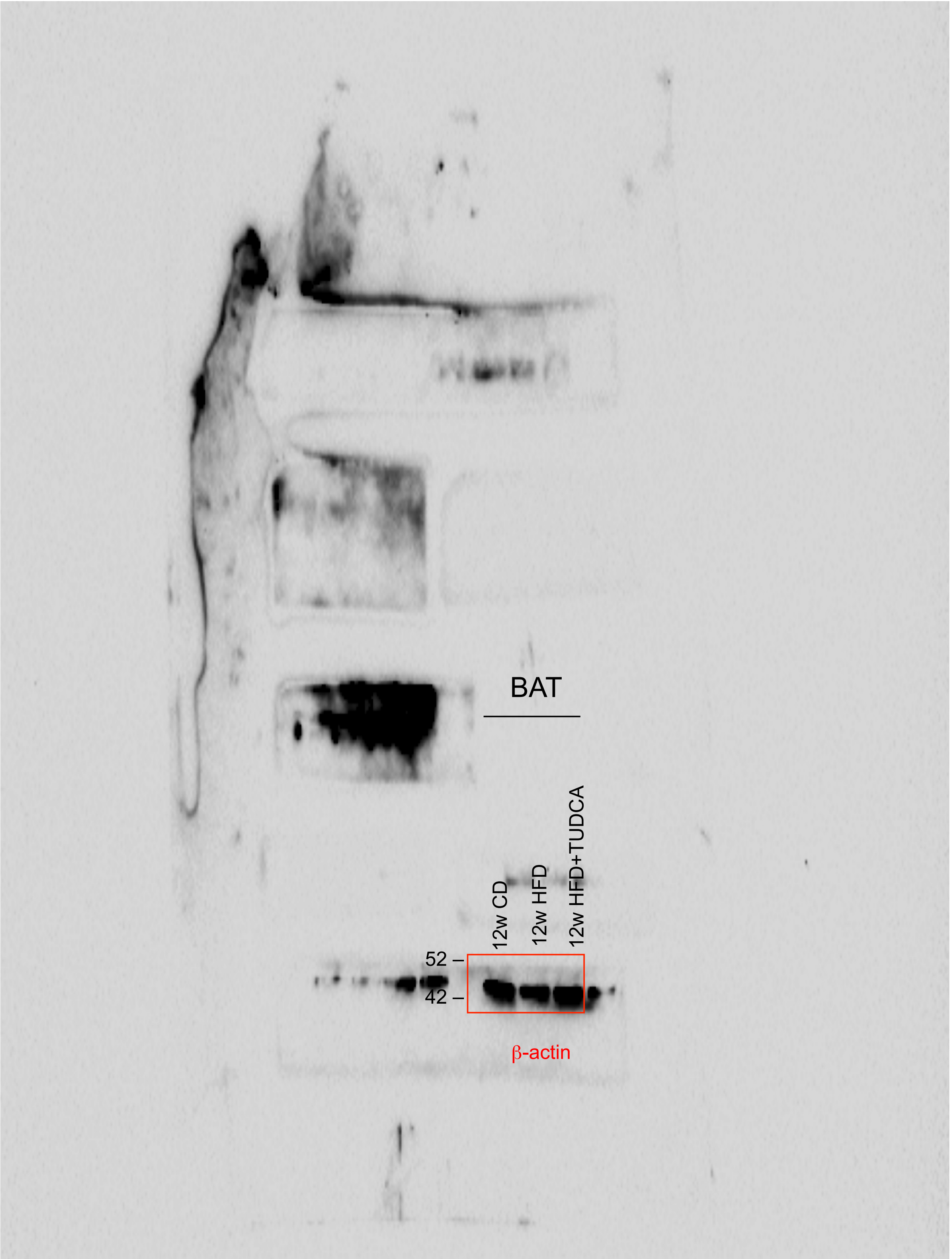

Uncut blot for Figure 5d

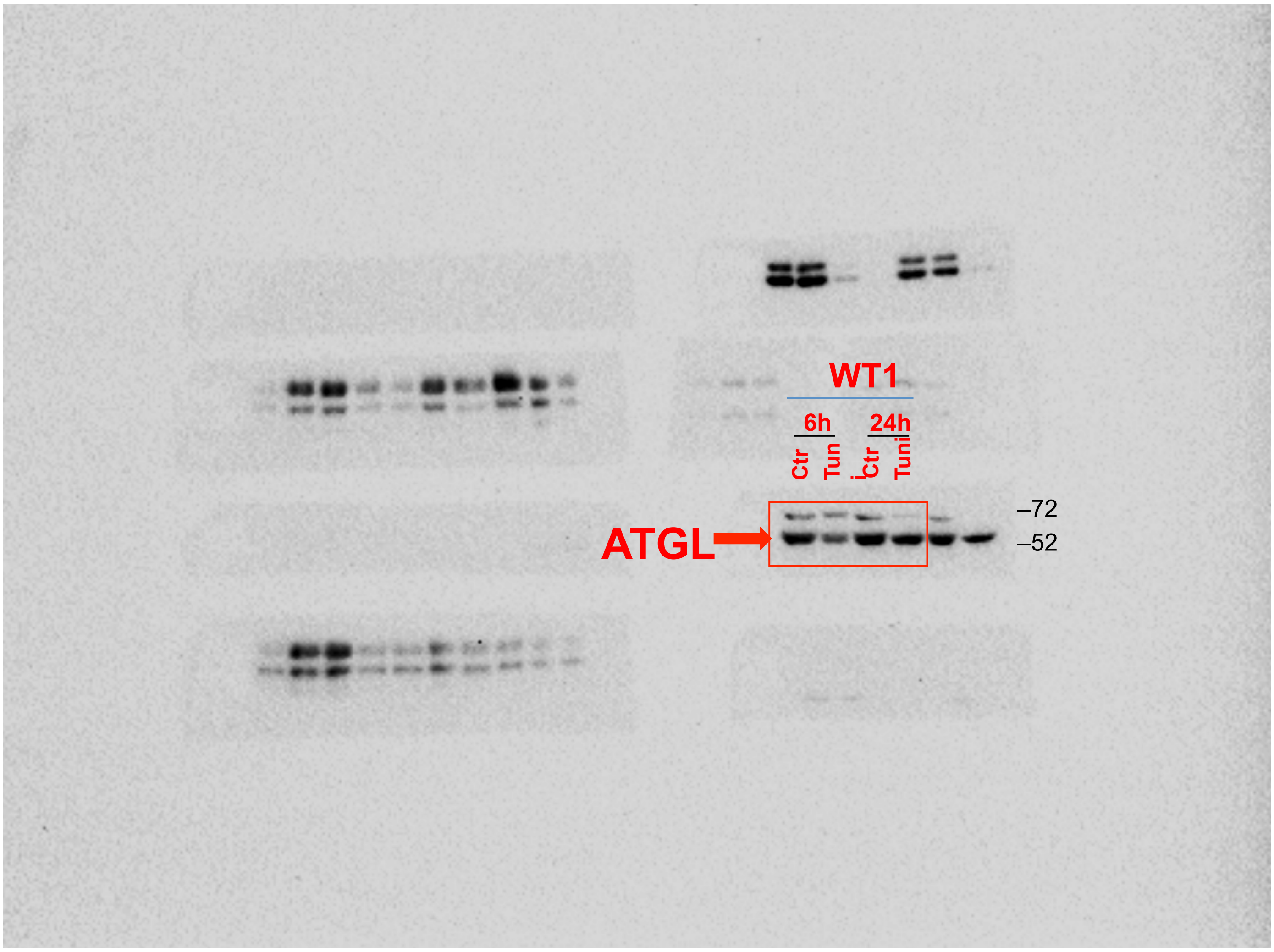

Uncut blot for Figure 6b

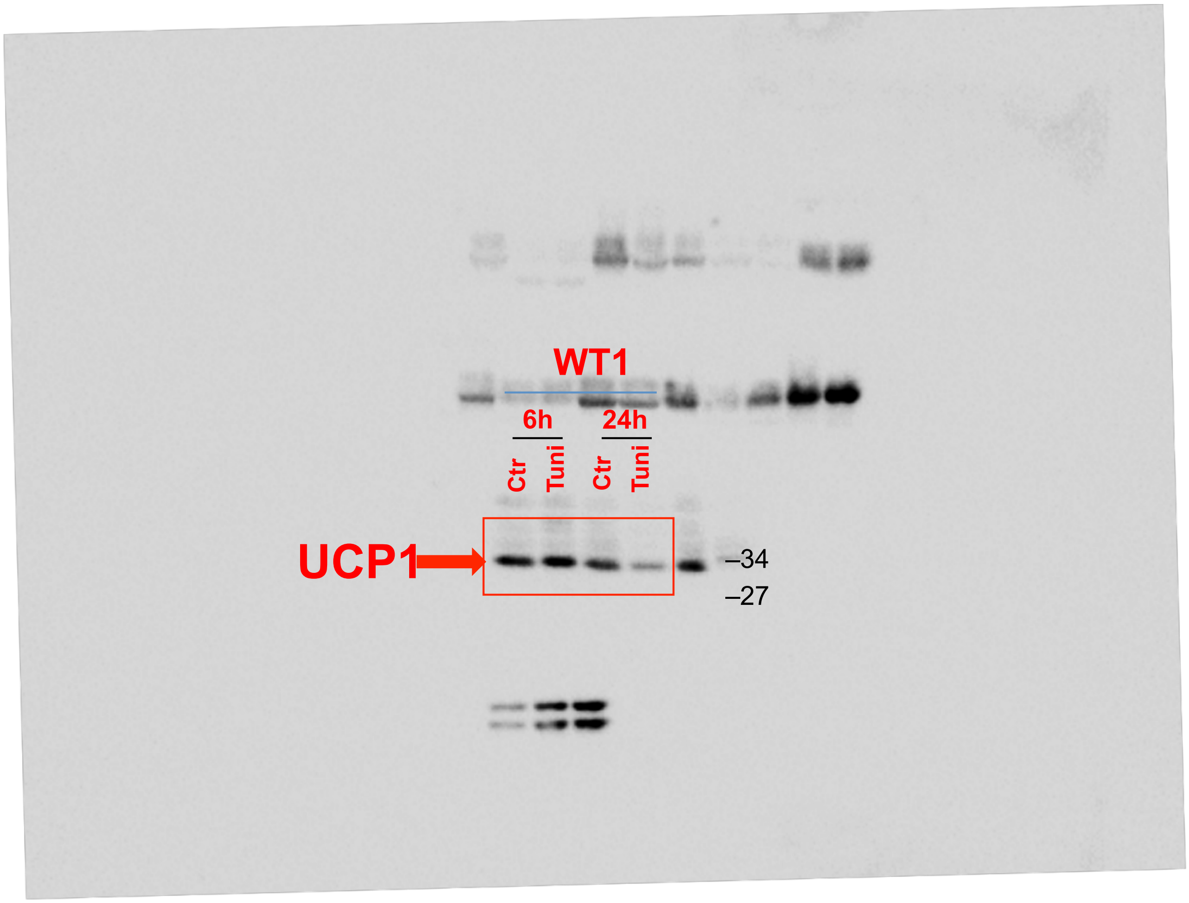

Uncut blot for Figure 5d & 6b

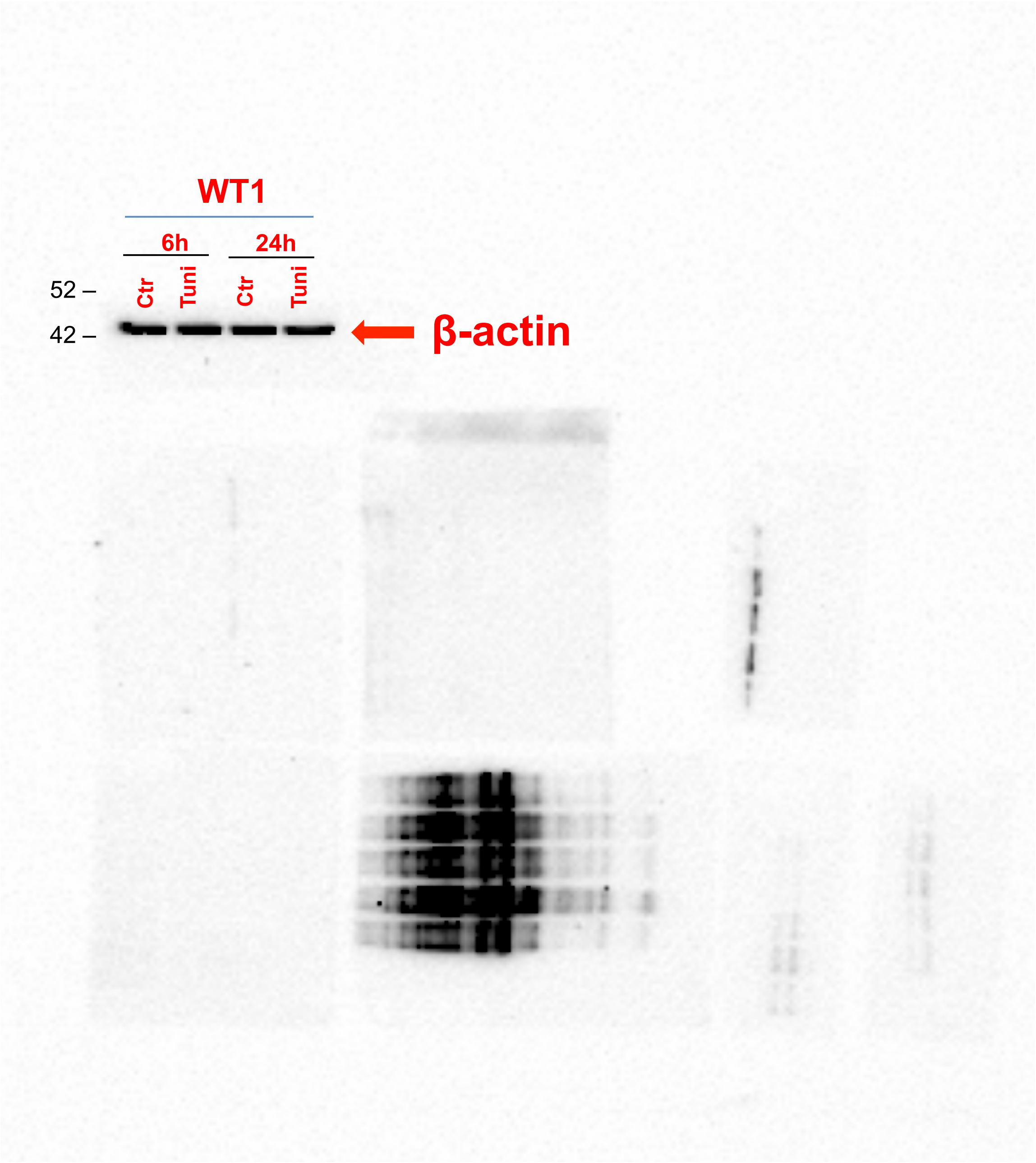

Supplement: Supplementary Information [file srep40215-s1.pdf]
